# Legal implications of the climate-health crisis: A case study analysis of the role of public health in climate litigation

**DOI:** 10.1371/journal.pone.0268633

**Published:** 2022-06-15

**Authors:** Narayan Toolan, Hannah Marcus, Elizabeth G. Hanna, Chadia Wannous

**Affiliations:** 1 UCLA School of Law, Los Angeles, California, United States of America; 2 World Federation of Public Health Associations–Environmental Health Working Group; 3 School of Public Health, University of Alberta, Edmonton, Alberta, Canada; 4 Australian National University, Fenner School for Environment and Society, Canberra, Australia; 5 WG Chair, World Federation of Public Health Associations–Environmental Health Working Group; 6 Towards A Safer World Network and Future Earth Health-Knowledge Action Network, Stockholm, Sweden; Szechenyi Istvan University: Szechenyi Istvan Egyetem, HUNGARY

## Abstract

**Background:**

Strong scientific evidence affirms that climate change is now a public health emergency. Increasingly, climate litigation brought against governments and corporations utilizes international human rights, environmental and climate laws and policies to seek accountability for climate-destructive and health-harming actions. The health impacts of climate change make litigation an important means of pursuing justice and strategically challenging legal systems. Yet there is scant documentation in the literature of the role that public health has played in climate litigation and the legal weight public health narratives are given in such contexts. Therefore, we assessed to what extent courts of law have used public health harm in legal adjudication and sought to provide practical recommendations to address barriers to positioning legal arguments in public health-centric frames.

**Methods:**

We reviewed legal databases to identify all publicly reported, documented, cases of climate litigation filed in any country or jurisdiction between 1990 and September 2020. For the 1641 cases identified, we quantified the frequency of cases where health concerns were explicitly or implicitly raised.

**Findings:**

Case numbers are trending upwards, notably in high income countries. Resolution remains pending in over half of cases as the majority were initiated in the past three years. Cases were primarily based in climate and human rights law and brought by a wide range of groups and individuals predominantly against governments. About half of the decided cases found in favour for the plaintiffs. Based on this, we selected the 65 cases that were directly linked to public health. We found economic forces and pricing of health risks play a key role, as courts are challenged by litigants to adjudicate on the responsibility for health impacts.

**Conclusions:**

While courts of law are receptive to public health science, significant legal reform is needed to enhance leveraging of public health evidence in legal judgements of climate litigation cases. The integration of a public health mandate into a new eco-centric legal paradigm will optimize its potential to promote human well-being—the core objective underpinning both international law, human rights, and public health. Existing legal doctrines and practices can be enhanced to increase the weight of public health arguments in climate legal action and consequently ensure legal rulings in climate litigation prioritize, protect and promote public health.

## Introduction

Since its inception, environmental law has been relied upon as a means of preventing harm to the environment, thus enabling healthy human relationships with the environment. Escalating Climate Change impacts across the world is increasing potential for litigation to be brought against corporations, governments, and other parties by individuals and communities on the basis of current or future harm to their health and wellbeing. This risk of legal liability is a key factor in shaping climate-compliant economies whereby actors seek to protect assets, minimize risk and seek opportunities that are within the boundaries of legal regulatory frameworks.

As the field of climate litigation has evolved, so have the legal strategies used to prosecute claims of wrongdoing. Only in the last few years have cases emerged from all areas of law including corporate law, financial law, and human rights, as strategic tools in the pursuit of litigation against climate-disruptive actions by corporations, governments, and other parties. The rising trends in litigation have been monitored and recorded by various scholars [1, 2]. With growing evidence of the health impacts resulting from climate change, it is unsurprising that public health concerns now feature more strongly in litigation. Yet there is scant documentation in the literature characterizing the role that public health arguments have played in climate litigation and the legal weight given to public health narratives.

Despite solid scientific evidence and the efforts of many, global greenhouse gas emissions are not reducing [3]. Similarly, despite environmental protection laws, environmental damage is demonstrably escalating as ecosystems decay is generating a sixth mass extinction [4]. The human species cannot flourish amid sick ecosystems. Healthy environments and a stable climate are necessary for human survival. Their disruption directly harms human health.

In recognizing that climate change is unfolding as a health emergency that disproportionately impacts the most vulnerable, health professionals are urging for accelerating efforts to decarbonize [5]. Accordingly, the public health community has increasingly issued calls for a “public health focussed response” across prevention, mitigation, adaptation, and preparedness [6]. Central to this approach is the need to ensure that viable legal avenues for deterring, halting, and seeking justice for climate-disruptive, health-threatening actions exist. To support this objective, this review provides a comprehensive analysis of the role of public health in climate litigation and provides practical recommendations to address barriers to positioning legal arguments in public health-centric frames.

Climate Litigation reveals the ways in which the threats of litigation can shape the transition to an economy which is not based on environmentally-devastating anthropogenic activities. Therefore, there is an urgent need for individuals, groups, stakeholders, and consumers to bring litigation that is correlated to a public health concern. Although litigation is often a lengthy, stressful and costly process, it may force governments and corporations to prioritize planetary and human health over profit.

## Background

### Climate change and human health

Since its identification as a growing environmental threat, climate change has generated concern from a variety of disciplinary perspectives. Only relatively recently has the public health perspective gained traction within the broader climate policy dialogue.

There is now robust data to substantiate the claim that climate change significantly and negatively impacts human health both directly and indirectly through a series of complex, interacting social and environmental determinants. This climate-health nexus featured strongly in the IPCC’s Fifth Assessment Report (AR5) [7] and was further expanded in the Working Group II contribution to AR6, Climate Change 2022: Impacts, Adaptation and Vulnerability [8].

Climate-driven health threats have taken the form of rising food [9, 10] and water [11, 12] insecurity, deteriorating air quality [13, 14], new infectious disease outbreaks [15–17], and fatal and disease-inflicting floods [18], droughts [19, 20], coastal storms [21, 22], wildfires [23, 24], heat waves [25, 26], as well as ecological grief and disaster-induced trauma which drive mental health illnesses worldwide [27, 28]. Intensifying climate-related disasters and food and water shortages also have broader, indirect consequences on human health, as mediated by their effects on patterns of conflict, forced displacement, and migration [29–31].

This ongoing “climate-health crisis” is expected to contribute further to rising global morbidity and mortality and social and economic suffering in the years to come [32]. Yet as stated in the Lancet Commission on the Legal Determinants of Health, “While law can be a powerful tool for advancing global health, it remains substantially underutilised and poorly understood. Working in partnership, public health lawyers and health professionals can become champions for evidence-based laws to ensure the public’s health and safety” [33]. Law is a powerful tool to advance global health because climate change adversely affects the physical health and mental health of people in all countries [34]. Present development challenges causing high vulnerability are influenced by historical and ongoing patterns of inequity such as colonialism, especially for many indigenous peoples and local communities [34]. Yet, according to the World Economic Forum “groups of stakeholders (the private sector, corporate and financial organizations) have taken part in a system that funds the destruction of nature” [35]. This is enabled by a rule of law, which more often gives primacy to corporate rights over the health of nature. Using scientific evidence, namely climate science and public health, law can advance health, human rights, and the rights of nature.

### Climate law, policies and economic incentives

Climate laws and policies are concerned with ‘mitigating, regulating or adapting’ industrial activity which causes climate change. The United Nations Framework Convention on Climate Change (UNFCCC) was signed by 154 states in 1992 acknowledging “that the global nature of climate change calls for the widest possible cooperation by all countries and their participation in an effective and appropriate response” [36]. Both the Paris Agreement [37] adopted by the Conference of the Parties (COP21) and the “UN Sustainable Development Goals (SDGs)” have guided development in this field. Although, health is not always specifically articulated in national laws and policies that address economic regulations on energy, electric utilities, construction, agriculture and industry, it is commonly implicit that the objective of these policies is human and planetary ecosystem survival. The evidential failure of *implied* protection of planetary health, stability of the climate and human health through existing laws provides a powerful argument that such protections must be made *explicit* and legally binding. For most nations the legal basis exists as in 2015, 193 nations endorsed the SDGs including SDG 3, which aims to “ensure healthy lives and to promote well-being for all ages” [38] and the Paris Agreement, endorsed by 196 countries, emphasizes the “right to health”. Whereas the SDGs are not legally binding, the Paris Agreement is. [39]. Yet with few legal teeth, it does not impose penalties, such as fees or embargos, for parties that violate its terms, and there is no international court or governing body ready to enforce compliance. Experts argue that in reality, the Paris Agreement is not legally binding [40].

The emerging field of climate law is inter-disciplinary and extends to often unsatisfactorily defined concepts such as a ‘carbon net zero economy’ and ‘sustainable finance’. Since 1992, the United Nations Environment Program (UNEP) Finance Initiative (FI) has partnered with the global financial services sector including banks, insurers, and investors in a commitment that “we … recognize that economic development needs to be compatible with human welfare and a healthy environment” [41]. Yet whilst older environmental regulations have provided legal avenues to petition for public health justice, they have been hindered by the economic ideology that profit trumps health, ecology, and ecosystems [42].

### Public health, the environment, and law

#### Environmental health-related legal frameworks

A relevant legal framework with implications for environmental health promotion is the International Environmental Law (IEL). IEL is concerned with the attempt to control pollution and the depletion of natural resources within a framework of sustainable development [43]. The resource management and pollution control components which comprise this framework make it inherently linked to public health objectives, despite there being no explicit mention of human health in many IEL statutes [44]. In October 2021, the United Nations, Human Rights Council (HRC), recognized that having a clean, healthy, and sustainable environment is a human right, however this is not a legally binding obligation [45].

Relevant IEL statutes include “The Declaration of the United Nations Conference on the Human Environment” (the 1972 Stockholm Declaration) [46] and “The Rio Declaration on Environment and Development” (the Rio Earth Summit) [47]. Inherent in both statutes is the legal mandate to prevent, wherever possible, the degradation or depletion of environmental resources needed to sustain planetary life. In both declarations, one can observe an anthropocentric focus on human health and the prevention of environmentally destructive actions which would be detrimental to it, namely those which interfere with life sustaining ecosystem services, as defined by the Millennium Ecosystem Assessment [48].

International Human Rights Law (IHRL), like IEL, features an implicit focus on human health and well-being, albeit through the imposition of protective policies against abusive acts which could threaten any one of its many sub-domains. There are notable areas where IHRL and IEL intersect to promote a policy environment conducive to human health. For example, climate-friendly policies are increasingly being linked to our moral duty to uphold Article 3 of the Universal Declaration of Human Rights [49] which states that “Everyone has the right to life, liberty and security of person”—rights which climate destructive actions explicitly threaten. Yet “environmental law continues to struggle with the complaint that it re-articulates some of the patterns of colonial exploitation in environmental terms”, most notably by treating nature’s resources as mere commodities and by failing to provide an enabling framework for indigenous populations to regain rightful stewardship over their lands [50]. Hence, reconciling environmental and human rights law remains an ongoing process, one which will arguably be central to creating a health-friendly legislative environment in the years to come.

On a national level, Environmental Law consists of statutes, regulations, applied common law, and in some countries, constitutional provisions. Currently, 155 countries have binding legal obligations to respect, protect and fulfill the right to a healthy environment [51]. Environmental rights consist of procedural rights (e.g. access to information, access to justice, right to participate in environmental decision making) and substantive rights (i.e. civil and political, economic and social, cultural, and collective rights) which implicate legal requisites to preventing environmental degradation [51].

Mapping the landscape within which environmental health features in international law also requires an understanding of International Health Regulations (IHR) which, in principle, embody a legal commitment of governments to respond to environmental health threats. The IHR are legally-binding for all 194 WHO member states and include requirements to report public health events with potential to cross borders and to maintain core capacities for preparedness, surveillance and response [52]. Consequentially, it can be said that obligations under the IHR imply a commitment to sound governance in response to environmental health emergencies which, by nature, have high potential to transcend international borders.

### Climate change litigation

Litigation is defined as “the act, process, or practice of settling a dispute in court of law” [53]. What is often disputed in climate litigation, is whether governments or market actors are ‘mitigating, regulating or adapting’ industrial activity which causes climate change. According to the IPCC [54], system change is required across all industrial sectors to substantially reduce greenhouse gas (GHG) emissions. Litigants and lawyers use such statements to support litigation and in recent years, individuals and communities have aligned their causes of social, human and economic justice with environmental and climate justice. Indeed, the Paris Agreement notes “the importance for some of the concept of climate justice, when taking action …” [37].

Legal Liability is defined as the *“responsibility that someone has for their actions*, *for example the responsibility to pay another person for harm or damage that is the result of these actions”* [55].

Climate laws and policies create new legal liability for market actors, but there is increasing evidence that polluting industries impose health burdens on their neighbors and that these risks are higher for black, brown, indigenous and poor communities [56]. As indicated in the cases discussed below, liability is sought not only using climate statutes, but using all areas of the law including corporate, financial, constitutional, and human rights.

Given the empirical grounding of CCL, climate attribution science is now playing an increasingly important role. By adopting epidemiological methodologies of quantifying proportional risk, climate attribution science “aims to establish the relationship between anthropogenic emissions and specific extreme weather events” [57]. As climate science has matured in the past decade, climate attribution methodologies are now increasingly capable of providing evidence to demonstrate causation and responsibility. By calculating the strength of the causal relationship, attribution studies bolster empirical arguments underpinning legal claims by allowing for evidence on proportional responsibility. The UNEP 2020 global status review on climate litigation suggested that climate attribution is being given more weight in the consideration of cases and determination of legally enforceable outcomes [58].

There are several types of detection and attribution studies focusing on: trends or long-term changes in climate variables; changes in extremes; weather or climate events; climate-related impacts; and the estimation of climate sensitivity using observational constraints. Detection and attribution studies can be done at various scales ranging from regional to global [59].

CCL includes both (a) cases where the claim is based on an established law, for which compliance/enforcement is sought, and (b) cases where there is no established law, but claimants assert that there ought to be (e.g. through expanding the scope of existing constitutional mandates, human rights statues, common law principals, etc). CCL has often been pursued through the international legal pathways of multilateral institutions and the United Nations system, drawing upon such doctrines as The Convention on the Rights of the Child, OECD Guidelines for Multinational Organizations, and the International Covenant on Civil and Political Rights, amongst others.

In the European Union (EU), litigation has often been orchestrated via reference to the Charter of the Fundamental Rights of the EU in addition to other relevant national/ international laws [60]. In the United States, CCL has been brought at both federal and state levels [47]. Most CCL has been against governments, but cases have been increasingly against corporate entities in relation to a widening array of financial, company, and corporate planning laws [61]. With growing requirements for corporate adherence to Environmental and Social Governance Standards (ESG’s), litigation against corporations is likely to increase in frequency in future.

Interestingly, eco-centric law is becoming a reimagination of the anthropocentric foundations of the international legal system. Eco-centric concepts are transforming human-centered legal systems, that historically undervalue non-human life. This is based on newly articulated eco-centric ideas that “to treat nature as just one of several competing (economic) interests results in a tendency to trump more qualitative public interest notions, such as ecosystem protection, intergenerational and intragenerational equity and even cultural values” [62]. This represents a profound departure from an anthropocentric framing of the environment as a mere resource-giving vehicle for human exploitation towards viewing the environment in Kantian terms, as “an end in itself”, as a legal entity with rights of protection.

Eco-centric concepts now extend to “wild property law”, ascribing personhood to “Eco-entities” in law, and laws of “Ecocide”. The French government is the first country to pass a law of Ecocide (2020) [63]. Despite some commentators saying this is a diluted form of the citizens’ convention recommendation, this is another indicator of the increasingly eco-centric orientation of recent legal developments. As legal developments are stimulated by this normative shift towards eco-centricity, a growing focus for public health stakeholders will pertain to the incorporation of a public health mandate into this new eco-centric legal paradigm. Will eco-centric laws be grounded in the One Health model [64] championed by environmental health practitioners and others? Will they respond to our need to reconcile goals of societal development, public health, and ecological preservation? It is these questions at the intersection of law and public health which will be important to address in future interdisciplinary collaborations.

Eco-centric laws have been passed in countries including: Ecuador in 2008 [65], Bolivia in 2010 and 2012 [66] (Law of the Rights of Mother Earth and Law of Mother Earth) and Panama in 2022. Panama grants nature the “right to exist, persist, and regenerate its life cycles”, defined as a “unique, indivisible and self-regulating community of living beings, elements and eco-systems interrelated to each other that sustains, contains and reproduces all beings” [67]. Other eco-centric laws have been codified in Colombia, Mexico, Uganda, and are included in Chile’s proposed constitution. In the United States, rights of nature have been shaped at the local level in more than 30 instances, including in the states of Pennsylvania, Florida, Minnesota and Colorado.

Giving rights to nature is a way to fulfill the human right to a clean, healthy and sustainable environment, as they are inter-dependent. Ultimately, both public health promotion and legal reform have common aims of creating a policy landscape conducive to human well-being. The integration of a public health mandate into a new eco-centric legal paradigm will optimize its potential to promote human well-being—the core objective underpinning both international law and public health. By knowing to what degree public health has featured in the empirical arguments used to justify climate legal action to date, we can better understand how well existing eco-centric and other environmental health laws lend themselves to public health promotion. Should gaps be identified, we can begin to postulate how existing legal doctrines and practices can be enhanced to increase the credibility of public health arguments in climate legal action and by consequence, ensure legal rulings on climate litigation have positive implications for public health.

## Objectives

The Grantham Research Institute on Climate Change and the Environment and Centre for Climate Change Economics and Policy examined in 2021 global trends in climate change litigation [68]. Our objective is to extend this work, by examining the extent, characteristics and success of cases based on protecting human health, either directly or indirectly by safeguarding environmental determinants of health.

Given a) the rising recognition of the human health threats posed by climate change and environmental devastation and b) the pre-existing and growing legal focus on protecting the environment (in both anthropocentric and eco-centric frames), on preventing infringement on the human right to health, and on regulating industry to protect public health, we set out to answer the following research questions:

1. In what ways has climate litigation evolved as a legal tool for climate action, and contributed to liability, particularly under the agenda of protecting the environment, safeguarding human rights, and promoting public health?
2. How receptive have courts been to legal cases grounded on public health?
3. How have references to public health contributed to legal decision-making in climate litigation?

## Method

We identified databases, with access to litigation documents, and judgements, of climate litigation, including translations from non-English languages. A review of climate science, legal databases was conducted on all such publicly reported, documented, English language (and translated) cases of climate litigation filed in any country or jurisdiction between 1990 and September 2020, to identify cases which relied upon public health science and can be categorized as public health climate litigation.

The following search terms were entered into available legal case databases: “climate change”, “climate change and public health”, “human rights and climate change”, “governments and climate change”, “corporations and climate change”.

We found litigation which was reported using the term climate change, or where it was implied by the subject via reference to relevant social issues, biodiversity loss, environmental degradation, carbon emissions, economic/land/property loss, linked legal regulation, or linked corporate governance. Cases were also found where climate change was peripheral to the argument such as where general environmental destruction was the primary concern, yet climate disruption was alluded to as a secondary consequence.

We extracted consistent, basic, pre-defined data from the cases documents on plaintiff, defendant, legal precedent, decision, core argument, and court, to assess cases with an implicit concern for health. We performed a search in these case documents to quantify the number of times that the word health was explicitly used in this litigation. We used the case data to identify climate litigation with a legal strategy, where the plaintiff relied on climate science and public health as part of a systematic challenge to a field of law. This resulted in 65 cases categorized as public health climate litigation, where public health was part of the litigation strategy.

The primary sources for this research have been the respective case law databases of the Grantham Research Institute on Climate Change and the Environment [68], and the Sabin Center for Climate Change law [69]. We also searched ECOLEX: a data base operated by FAO, IUCN and UNEP, whose purpose is to “build capacity worldwide by providing the most comprehensive possible global source of information on environmental law and includes treaties, treaty decisions, legislation, and literature along with jurisprudence”.

The primary databases are the most comprehensive, publicly available databases for researchers of case law in the field of Climate law. According to the methodology of the database provided by the Grantham Research Institute for Climate and the Environment: “The analysis covers all UN and UNFCCC parties, including the European Union, as well as a number of countries, regions and territories that are not UN or UNFCCC members (e.g. Taiwan, Palestine and Western Sahara)*”* [70].

The focus of our research is on the role of public health in strategic climate litigation, building upon the significant document pooling already contained in the chosen sources, including the translation of non-English language case documents. The databases of the Grantham Research Institute and the Sabin Center for Climate Change law are organized under a climate science agenda, with an interdisciplinary focus on climate change. Despite increasing literature on climate law, this is an emerging field, and there are limited data sources for such research.

While these databases were considered relatively robust sources for the comprehensive identification of past litigation cases, it is notable that they have some limitations in their scope, as discussed in greater detail in the limitations section.

## Results

### Temporal trends

The literature review yielded a total of 1641 cases of climate litigation filed between 1990 and September 2020. As of November 2020, a total of 2069 Climate Laws and Policies were in force [71]. The numbers according to region are: (i) East Asia and Pacific– 391 (ii) Europe and Central Asia– 677 (iii) the EU– 41 (iv) Latin American and the Caribbean– 364 (v) Middle East and North Africa– 125 (vi) North America (including Canada)– 33 (vii) South Asia– 109 (viii) Sub Saharan Africa– 383 [72]. Of these 2069 Climate Laws and Policies, 1920 (93%) have been passed since 2000, and 1367 (66%) have been passed since 2010 [73].

**Fig 1:**
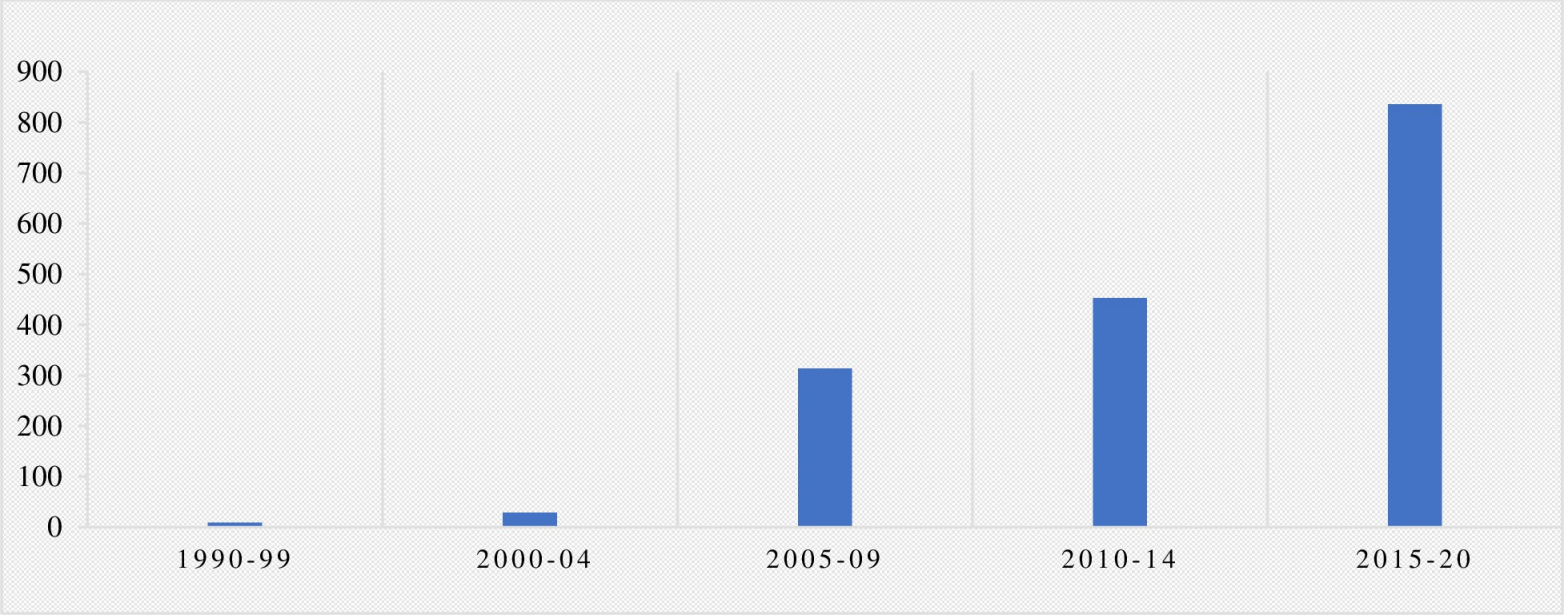
Total number of climate litigation cases by time period (1990–2020).

The distribution of these cases by year is shown in Fig 1 which depicts a clear trend of increasing numbers of cases across time, with only 9 cases filed from 1990–99 and 836 cases filed from 2015–20. Not shown in Fig 1, the year the Paris Agreement came into force was pivotal, with a sharp increase in cases after 2016.

Additional to an increasing trend in cases, we noted explicit references to climate change became notably more frequent in the post-2000 cases. In the 1990–99 cases, climate change was seldom explicitly mentioned as the core issue, and legal arguments primarily related to broader (though inherently climate-linked) issues of environmental harm and destruction, air and water pollution, and land-use change, amongst others. Anthropocentric conceptualizations of the environment as a human-serving/resource-producing asset were also notably more apparent in the 1990–99 cases.

In contrast, post-2000 cases featured far more explicit references to climate change. Not only were allegations grounded in an explicit focus on climate change, but many cases, particularly in more recent years linked human rights (including the right to health) with de-carbonization, sought increased liability for climate risk-associated financial/investment activity, as well as legal attribution of personhood for the environmental, or legal accountability to future generations impacted by climate change. Compatible with this finding is the temporal trend of increasing likelihood of case-driven policy change across time. Indeed, in keeping with accelerating climate change, most of the cases which directly led to the passage of new climate-friendly, environmental health-rooted, or human rights-oriented legislation or legal statutes occurred in the past 5 years. This trend suggests maturation of climate law.

### Characteristics of cases

**Fig 2:**
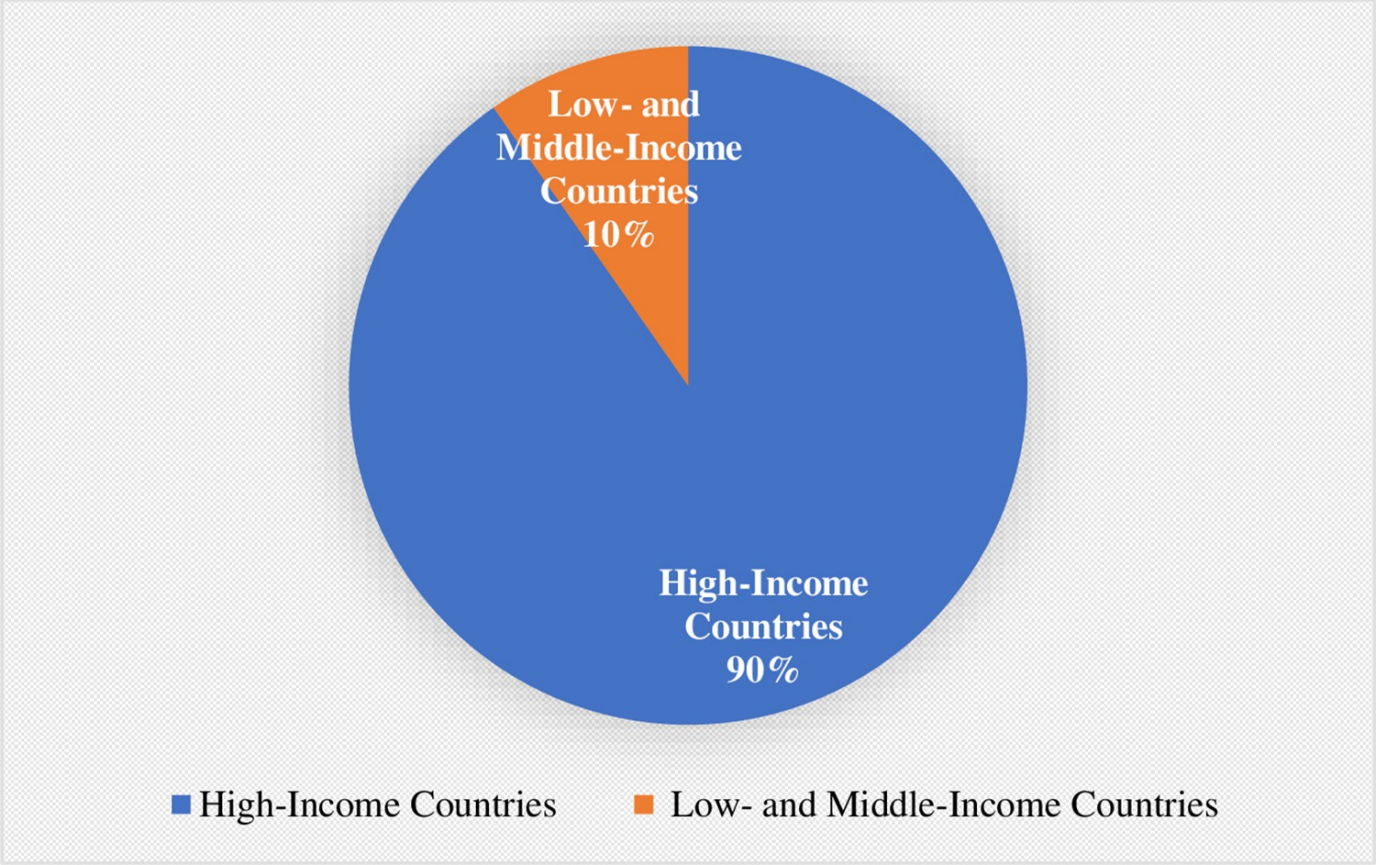
Distribution of non-USA climate litigation by World Bank development category.

Of the 1641 cases identified, the vast majority (n = 1241) were filed in the US, with only 360 filed elsewhere. The majority of the cases pursued outside the USA (90%, n = 325) were filed in high-income countries, with only 35 (10%) filed in low- and middle-income countries (LMICs) [74] (Fig 2).

**Fig 3:**
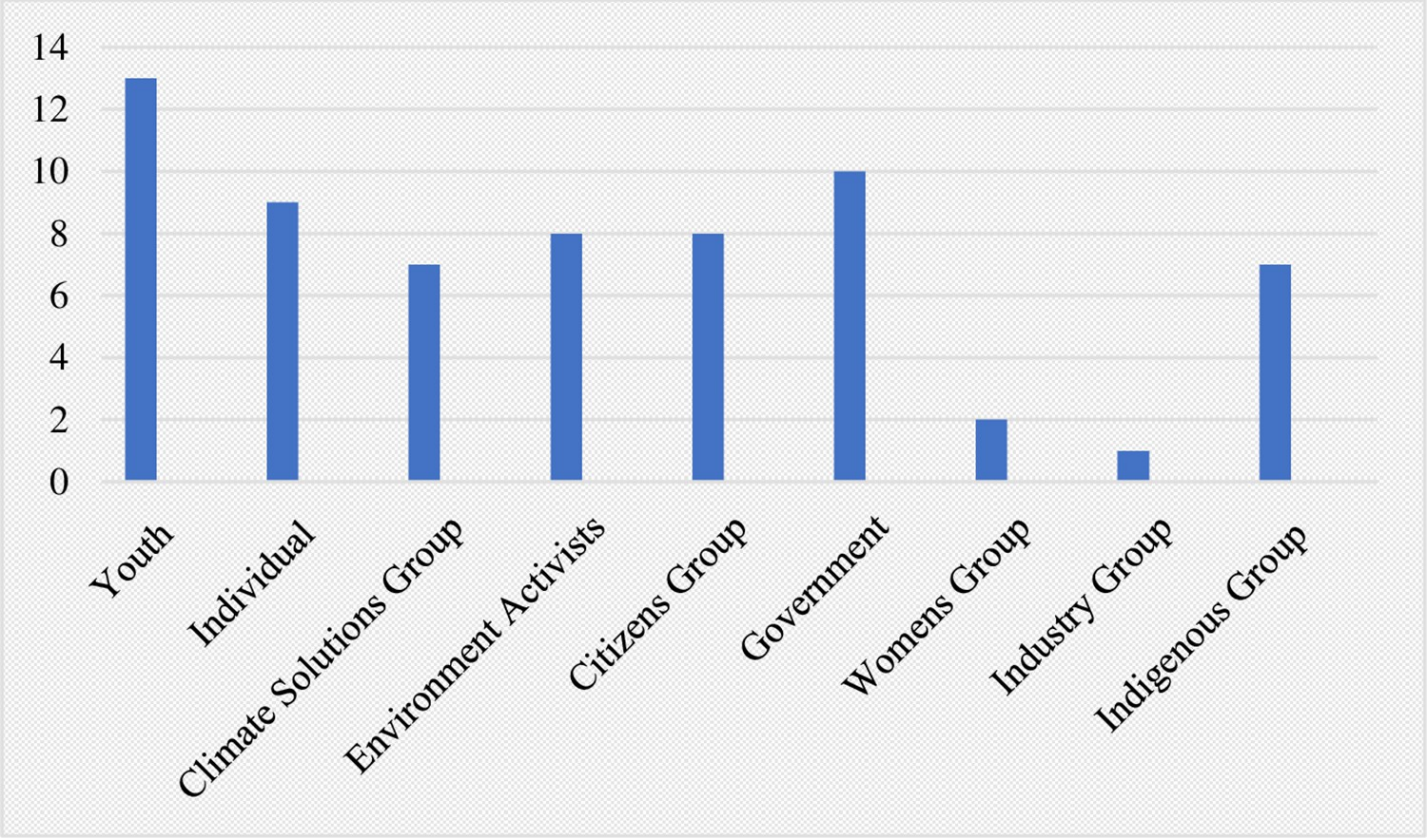
Public health-linked climate litigation cases by category of plaintiff.

**Fig 4:**
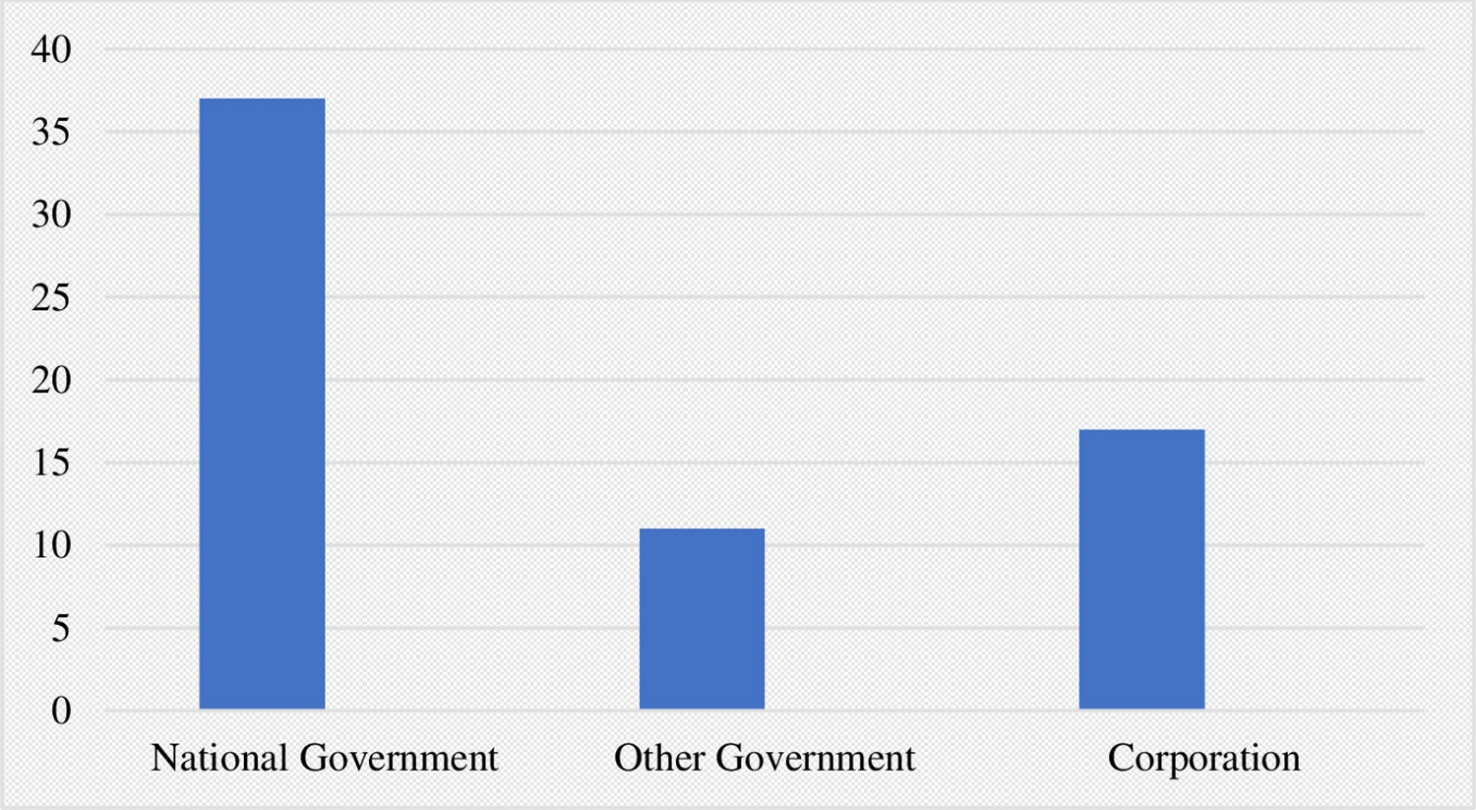
Public health-linked climate litigation cases by category of defendant.

The distribution of cases by plaintiff and defendant type (the actors against whom cases have been filed) for the 65 directly public health-linked climate litigation cases identified is depicted in Figs 3 and 4, respectively.

With regards to plaintiff type, youth comprised the largest category, followed by government then individuals independent of interest groups. The data concerning defendant type depict an overwhelming majority of cases against governments, with fewer filed against corporations. Nonetheless, when analyzing temporal trends across all cases (not only public health-linked ones), we observed the number filed against corporations increased greatly over the past two years, (7 cases in 2019 and 3 in the first half of 2020, compared to one case in all of 2015).

**Fig 5:**
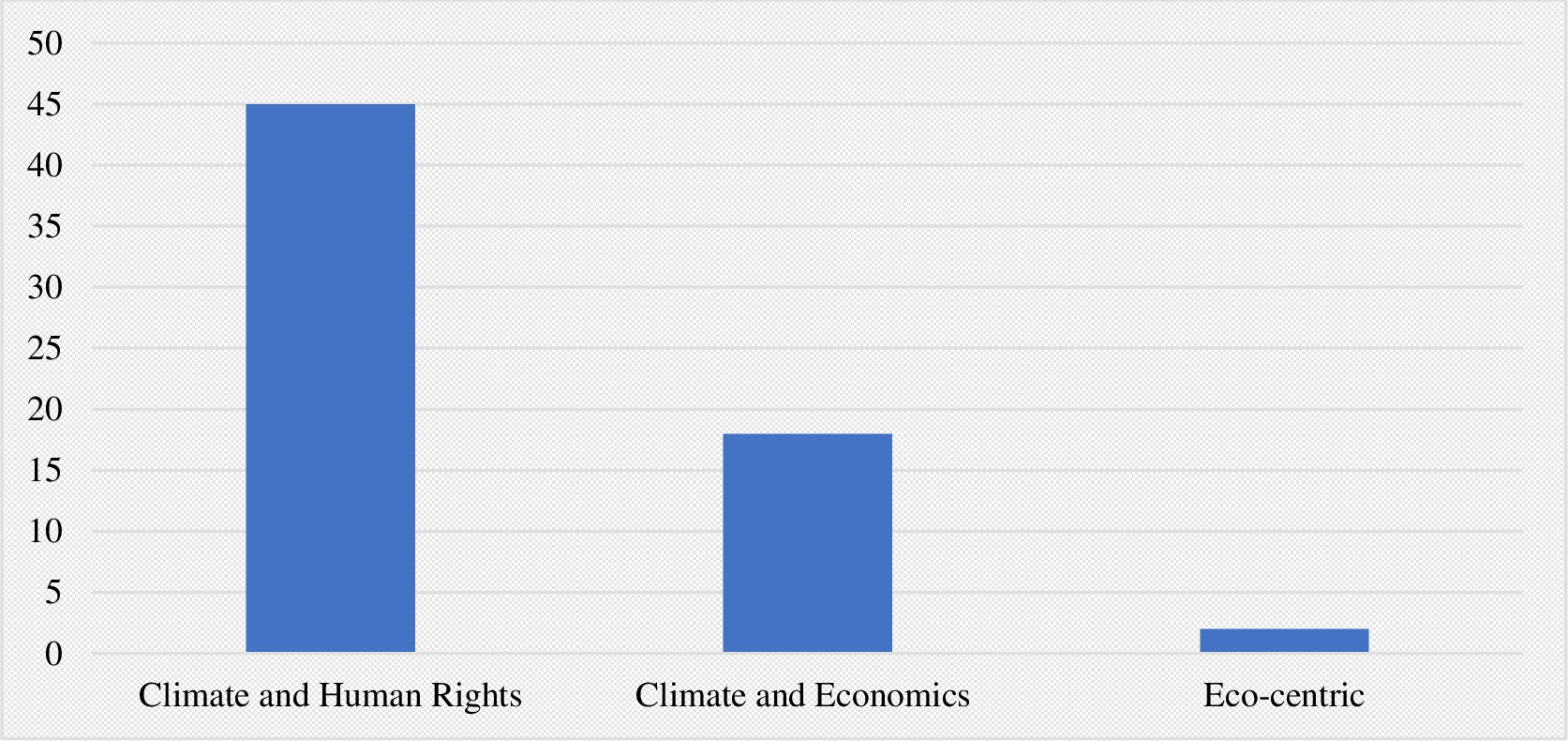
Public health-linked climate litigation cases by field of law.

Diverse legal claims and arguments were put forth in the 65 directly public health-linked cases, involving a complex distribution of legal precedents on which accusations of wrongdoing were based. The cases were categorized into human rights, economic, and eco-centric focused arguments. Human rights claims predominate, with most plaintiffs equating assaults on health with what are deemed to be clear human rights violations (Fig 5).

### Findings on public health-linked cases

**Fig 6:**
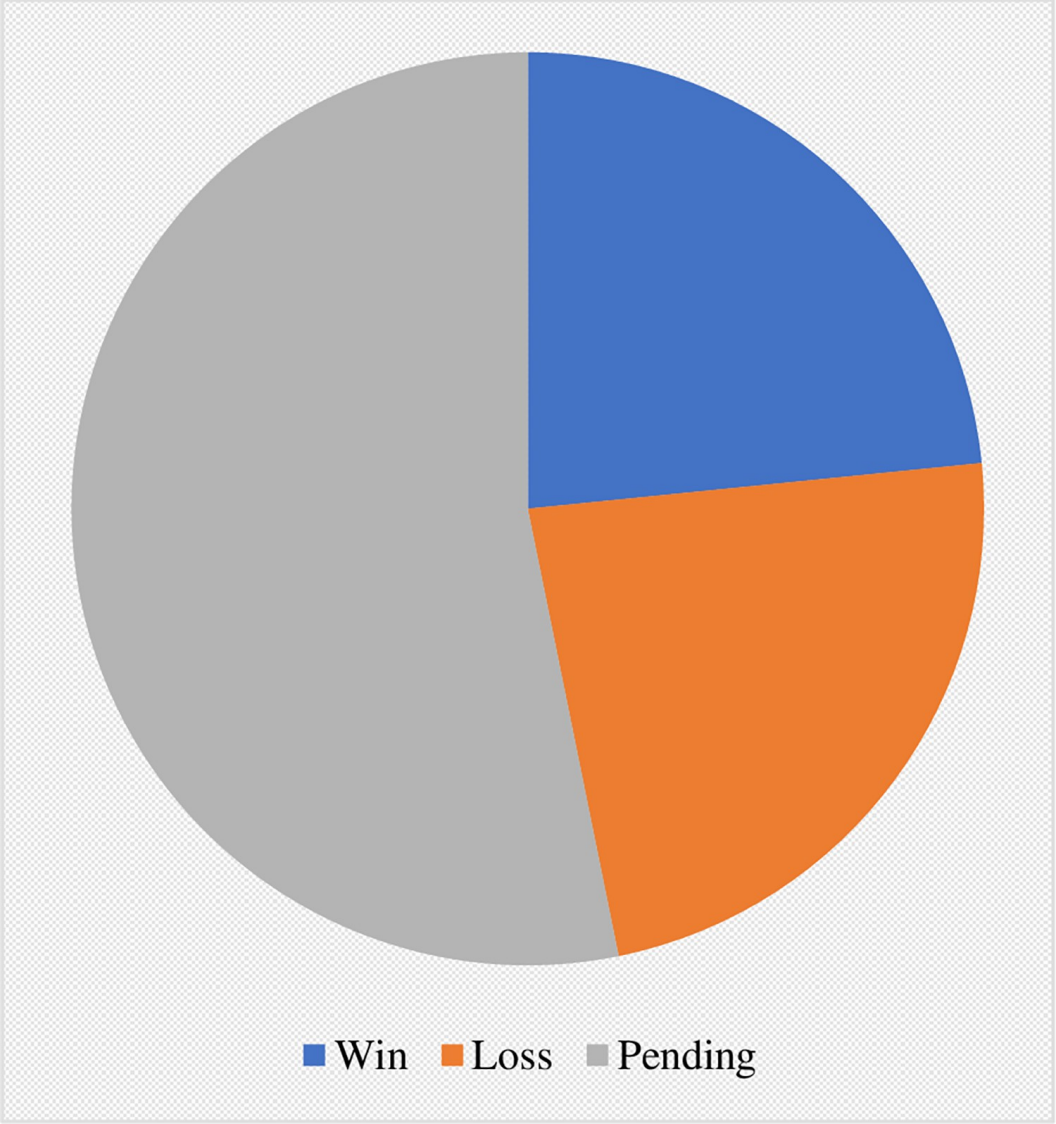
Current status of public health-linked climate litigation cases (April 2020).

**Fig 7:**
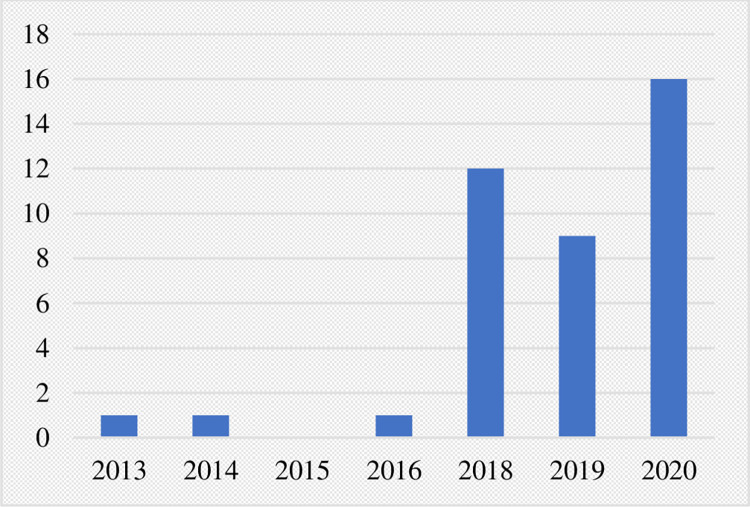
File year of pending public health-linked climate litigation cases.

**Fig 8:**
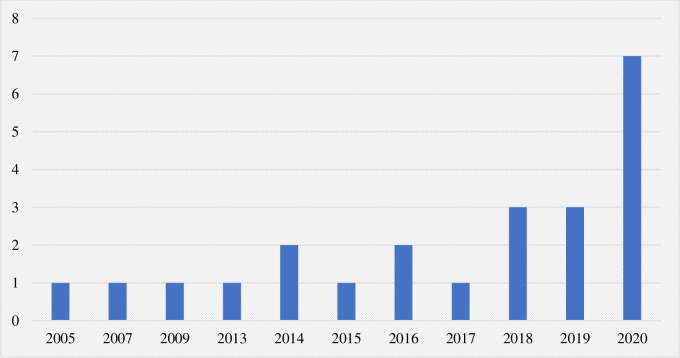
Verdict year of decided public health-linked climate litigation cases.

Of the 1641 cases of climate litigation identified in this review, only 65 were categorized as directly “public health-linked” on the basis of plaintiffs explicitly mentioning human health in legal argumentation and/or expressing a value for human health through emphasis on human health-environment interconnections. The status of these cases as of September 2020 is depicted in Fig 6, with 11 cases achieving success for the plaintiff, 11 cases determined for the defendant, and 25 cases still pending decision. The file years for pending cases and verdict years for decided cases are shown in Figs 7 and 8, respectively. In both, a clear temporal trend with rising frequency of both filed and decided cases is shown.

Nine notable cases that occurred in the past 5 years were selected for more in-depth analysis based on their strong public health linkages. These cases are listed and summarized in chronological order in Table 1, with indication of the number of times the word “health” (both human and environmental health) was used in the case document submitted to the court. The number of times the word “health” was explicitly used ranged widely from twice (case 6) to a maximum of 28 (case 4).

**Table 1:** Recent public health-linked climate litigation cases.

Case No.	Plaintiff v Defendant	Court and Year Case Filed	Brief Case Description and Outcome (If Available)	# Times Health Mentioned	Example of Health Term Usage
**1**	Urgenda Foundation v State of the Netherlands [75]	Netherlands Court of Appeals, 2015 (Decided 2019)	**Case:** Appeal by Urgenda Foundation for Dutch gov’t to reduce emissions by minimum 25% before 2020 **Outcome:** Court order for Dutch gov’t to adopt stringent policies to reduce emissions by 25% before 2020	4	*“This will result in*, *among other things*, *the significant erosion of ecosystems which will*, *example*, *jeopardise the food supply*, *result in the loss of territory and habitable areas*, *endanger* ***health***, *and cost human lives*.*”*
**2**	Future Generations v Ministry of the Environment [76]	Supreme Court, Bogota, 2018 (Decided 2018)	**Case:** Youth plaintiffs seek to enforce fundamental rights to a healthy environment which they claim are threatened by climate change and deforestation. **Outcome:** Court decided that the Amazon has personhood in law thus giving rights to nature	7	*“Fundamental rights of life*, ***health***, *the minimum subsistence*, *freedom*, *and human dignity are substantially linked and determined by the environment and the ecosystem*.*”*
**3**	Maria Khan et al. v Federation of Pakistan [77]	Lahore High Court, Pakistan 2018	**Case:** Lawsuit filed by Maria Khan and group of women against Pakistan gov’t for inaction on climate change, resulting in violation of fundamental rights to clean and healthy environment, climate capable of sustaining human life, equal protection for women under the law, and no discrimination on the basis of sex **Outcome:** Pending	7	*“The Impugned Conduct infringes the right to life and the right to dignity of the Petitioners*, *by violating their right to a clean and* ***health****y environment and a climate capable of sustaining* ***human life*.***”*
**4**	Neubauer et al. v Germany [78]	Federal Constitutional Court of Germany, 2020 (Decided April 2021)	**Case:** Lawsuit filed by Neubauer and group of German youth against German gov’t for failing to uphold national obligation under Paris Agreement to limit global temperature rise to “well below 2 degrees Celsius”; appeal for German gov’t to raise GHG reduction target from 55% to 70% by 2030 **Outcome:** The Court held that the legislature had not proportionally distributed the budget between current and future generations. Legislature ordered to set clear reduction targets.	28	*“Any increase in global temperature (e*.*g*. *+0*.*5°C) will above all have negative impacts on human* ***health****–this is undisputed in the scientific literature following the IPCC Special Report on 1*.*5°C (Annex 3)*.*”*
**5**	District of Columbia v Exxon Mobil Corp. [79]	District of Columbia Supreme Court, USA, 2020	**Case:** Lawsuit filed by the District of Columbia against oil and gas companies for allegedly violating the Consumer Protection Procedures Act by misleading consumers about “the central role their products play in causing climate change” **Outcome:** Pending	12	*“These events threaten human* ***health***, *food security*, *agriculture*, *economic productivity*, *water supplies*, *national security*, *and labor productivity*.*”*
**6**	Youth for Climate Justice v EU Member States [80]	European Court of Human Rights, 2020	**Case:** Lawsuit filed by six Portuguese youth against 33 EU Member States for violating human rights by failing to take sufficient action on climate change; appeal for 33 EU Member States to take more ambitious action **Outcome:** Pending	2	*“Resolution of the question of what constitutes a state’s ‘fair share’ in favour of the Applicants is vital if the objective set out in Article 2 of the Paris Agreement–of preventing “significant deleterious effects […] on human* ***health*** *and welfare” by limiting global warming to 1*.*5°C–is to be achieved*.*”*
**7**	Juliana v United States [81]	United States Court of Appeals for the Ninth Circuit, 2015 (decided 2020)	**Case:** In a split decision, the Ninth Circuit Court of Appeals ruled that young people and other plaintiffs asserting a claim against the federal government for infringement of a Fifth Amendment due process right to a “climate system capable of sustaining human life” did not have Article III standing. **Outcome:** Decided against the Plaintiff. Petition for re-hearing has been submitted.	9	*“In these proceedings*, *the government accepts as fact that the United States has reached a tipping point crying out for a concerted response—yet presses ahead toward calamity*. *It is as if an asteroid were barreling toward Earth and the government decided to shut down our only defenses*. *Seeking to quash this suit*, *the government bluntly insists that it has the absolute and unreviewable power to destroy the Nation” STATON*, *District Judge*, *dissenting against the majority ruling*.
**8**	UN Human Rights Committee Views Adopted on Teitiota Communicatio [82]	United Nations Human Rights Committee, 2015 (decided 2020)	**Case:** On September 15, 2015, Teitiota filed a communication with the UN Human Rights Committee, alleging that New Zealand had violated his right to life under the International Covenant on Social and Political Rights. The New Zealand Supreme Court had decided that the applicant did not qualify as a climate refugee under international human rights law. The Committee ruled that an arbitrary deprivation of life must be personal rather than rooted in the general conditions of the receiving state. **Outcome:**Case dismissed on merits.	21	*“It would indeed be counterintuitive to the protection of life*, *to wait for deaths to be very frequent and considerable; in order to consider the threshold of risk as met*. *It is the standard upheld in this Committee*, *that threats to life can be a violation of the right*, *even if they do not result in the loss of life*. *It is should be sufficient that the child of the author has already suffered significant* ***health*** *hazards on account of the environmental conditions”*, *dissenting judgement*.
**9**	Milieudefensie et al. v Royal Dutch Shell [83]	Netherlands, The Hague, District Court, 2019	**Case:** This case built on the landmark Urgenda decision which found that the Dutch government’s inadequate action on climate change violated a duty of care to its citizens. In the suit against Shell, plaintiffs extend this argument to private companies, given the Paris Agreement’s goals and the scientific evidence regarding the dangers of climate change.**Outcome:**Defendant Shell must reduce carbon emissions by 45% by 2030 relative to 2019.	77	*“Humans depend on* ***healthy*** *and sufficiently vital ecosystems for their lives and well being*, *that can provide such ecosystem goods*, *functions*, *and services that humans need for their existence*, *in a sufficiently reliable manner*. *Climate change is a threat to ecosystems and therefore a threat* ***to human life*** *and well-being”*

## Discussion

### The evolution of litigation as a legal tool for climate action

The recent surge in climate litigation, characterized by a notable uptick in cases filed since 2015, is testament to the growing recognition of the potential value of using litigation as a tool for climate action. The rising trend in cases filed (Fig 1) indicates a growing expectation that actors ought to be held accountable for actions which wreak havoc on the environment and threaten the prospect of sustaining a climate compatible with human life. These temporal trends are generally consistent with findings from other studies which have documented a relatively consistent rise in both US- and non-US-filed climate litigation cases [84].

The discourse on environmental health now occurring in legal courts can also be said to represent an increased appreciation for the role of Ecosystem Services in supporting health and well-being, not only in terms of material resources (“provisioning services”) but also sheltering populations from physical harm for example purifying water (“regulating services”), maintaining planetary equilibrium (“supporting services”), and promoting spiritual and cultural value (“culturally enriching services”) [48].

The post-2015 surge in cases has been linked with two major 2015 events: 1) the passage of the Paris Agreement which solidified national commitments to carbon emission reduction to limit global warming to well below 2, preferably to 1.5 degrees Celsius, compared to pre-industrial levels, providing more legal leverage for plaintiffs suing purely on the basis of GHG emission exceedances [85], and 2) the landmark judgement delivered in the *Urgenda* case where a class-action brought by 886 Dutch citizens resulted in a court order for the Dutch government to adopt a more ambitious emission reduction target [86].

The momentum created by the success of *Urgenda* (Table 1, case 1) led to similar cases emerging in the domestic tribunals of several jurisdictions all over the world, ranging from Belgium to France and South Korea, beginning what has been referred to as the “new wave” of climate litigation [86].

Despite the trend in plaintiffs using litigation as a legal tool for climate action, it remains unclear, from our results and other studies [84], to what extent climate litigation generates new government legislation or policy. This uncertainty is especially pronounced among LMICs, where the frequency of filed cases remains extremely low, representing only 2% (n = 35) of the 360 non-US cases. Notwithstanding the relatively high volume of litigation, North America (including Canada) has passed only 33 climate laws and policies, which is significantly less than the 677 passed in Europe and Central Asia. Whilst outside the scope of this study, analyzing the relationship between existing legal frameworks, breaches of these and numbers of cases brought before courts is an area worthy of further research.

The total rise in cases is due largely to the recently documented increases in annual litigation occurring in the US and in a select few other jurisdictions including Australia, New Zealand, the European Union, Spain, and the United Kingdom [87]. However, litigation may indicate that there are political, cultural or other obstacles to climate justice in a particular jurisdiction. In *Juliana v United States* (Table 1, case 7), despite a 38-page submission by leading public health experts stating that “without significant intervention, this new era will come to define the health of an entire generation”, the court declined to directly adjudicate on climate change. The court explained the adjudication did not refute the science, rather recoiled from passing a political ruling in the following statement: “There is no justiciable ‘controversy’ when parties seek adjudication of a political question… It is not the province of the judiciary to make the policy decisions required to grant Plaintiffs the relief they seek. Nor may this Court direct other branches of government to overhaul their fossil fuel, agriculture, logging, and family planning policies to address climate change. To suggest otherwise is to overlook ‘the separate and distinct constitutional role’ of the judiciary” [81]. This ruling may yet be overturned on appeal, but the judges of this case interpreted the constitution so as to hold that climate policy must arise from the legislative and political process, and not the judiciary.

Scholars have highlighted that the role of the global South in climate litigation should not be overlooked. We find this contention meritorious because, despite the relatively low numbers of cases from LMICs, many, most notably in Colombia, Ecuador, Bangladesh and India, were eco-centric cases brought against powerful companies. The important context here is that the 52 LMICs produced a fraction of the total 2020 CO_2_ emission [88], yet, they disproportionately bear the brunt of climate change impact and a higher risk of climate-driven health threats. Therefore, it is particularly salient for LMICs to use legal arguments based on climate injustice and vulnerability.

Finally, our finding that a growing number of cases are being brought by individuals (for instance as class-action lawsuits) provides further evidence of an increasingly “bottom-up approach to climate governance” [86]. From a pro-health standpoint, this can be viewed as an indication of rising public awareness of the threat of climate change and an accompanying realization by individuals and civil society of their democratic right to hold corporations and governments accountable for actions which infringe their rights to a healthy environment. Our analysis of litigation outcomes shows marked heterogeneity. Increased pressure on legislatures and corporations to change their behaviour is clearly evident, yet many courts remain slow to take the fullest action that the science calls for. Trends suggest this pressure is likely to increase this decade.

Still, early successes particularly in the field of human rights give reason for optimism; amassing a folio of successful litigation that translates to pro-health and pro-environment policy can be expected to motivate others to bring forward cases citing egregious occurrences or prospects of harm.

### Temporal and geographic trends in legal argumentation

#### Climate change as a peripheral vs. core issue

Climate Change Litigation (CCL) has gained prominence over the past three decades as a “governance mechanism for addressing climate change” [61]. In the late 1980s and early 1990s in the United States, climate legal action began as a means to hold actors accountable for activities knowingly contributing to GHG emissions exceeding national guidelines [61]. Our database searches identified that between 1986 and the end of May 2020, a total of 1,587 cases of CCL worldwide had been brought before courts, with 1,213 (76%) cases in the United States and 374 cases in 36 other countries and eight regional or international jurisdictions. Out of this total we found 65 directly linked public health cases. With the mounting climate crisis unfolding, CCL continues to be a growing field of law that intersects International and National Environmental Law, International Human Rights Law, and a complex range of statutes, regulations, and legal principals spanning national, subnational and, in some countries, constitutional law.

One of the most notable temporal trends we observed was the increasing frequency of climate change being framed as the core issue. Litigation is evidently evolving as a specific tool for climate legal action. Early cases tended to present environmental destruction as the central concern and framed climate change as a peripheral or secondary issue. The 2020 climate litigation snapshot found climate change to be “at the centre of the legal argument” in about 41% of cases, whereas in the remaining 59% climate change was classified as a “peripheral issue” [61]. The increasing discussion of climate change as a core issue suggests the ‘legal mainstreaming’ of climate change, whereby the adaptation, mitigation, and regulation of industry, becomes a matter of business and financial law, discussed in detail below.

#### The changing role of environmental and human rights law in litigation

Our results confirm reported trends attesting to a substantive rise in the contributions of environmental and human rights law to legal argumentation in litigation.

Regarding human rights, several powerful case studies illustrate a trend towards petitioners increasingly employing rights claims in climate change lawsuits. In the *Urgenda* case (Table 1, case 1), the courts decided that human rights, which include the “right to health” is linked with decarbonization. In a case brought by *Friends of the Irish Environment* [89], the courts held that Ireland’s national mitigation plan partially violated statutory law, because it was not set to reduce GHG emissions sufficiently over the near term. In this case, human rights were used as part of the justification.

In the case of *Maria Khan et al*. *v the Federation of Pakistan* (Table 1, case 3), rights to life, dignity, and a clean and healthy environment were argued to be under threat by the actions of the defendants. Likewise, the constitutionally guaranteed “Right to Life” is evoked in the legal argumentation of *Future Generations v Ministry of the Environment* (Table 1, case 2), as is “*Article 2(2) of the Basic Law*: *Right to Life and Physical Integrity*” in the lawsuit filed against *District of Columbia vs*. *Exxon Mobil Corp*. (Table 1, case 5). In their analysis of other rights-based claims in climate litigation cases, Peel and Osofsky (2017) conclude that receptivity of courts to rights-based framing is on the rise because it provides a “tangible legal framework for analyzing state actions that lead to climate change” [90]. This is encouraging from a public health standpoint, as all UN human rights treaty bodies now recognize individual rights to life, health, food, and water [49, 91], providing a stronger legal avenue for plaintiffs to link claims of health threats to rights-based legal frameworks.

Human rights have also been used by the defense in ‘just transition litigation’ to question the “benefits and burdens of the transition away from fossil fuels and towards net-zero emissions” [92]. Public health science can provide critical evidence to ensure that human rights are protected, defended and upheld. That defensive interests do not succeed in using a human rights narrative, when it is corporate rights that are at stake, and not human well-being.

In relation to rights for nature, we also observed a rise in eco-centric framings of the environmental. This included the attribution of personhood to nature in some of the more recent cases and the recognition of the inherent value of the environment independent of ecosystem services.

In the case of *Future Generations v Ministry of the Environment* (Table 1, case 2), a group of youth plaintiffs in Colombia sued the government, municipalities and several corporations based on a “right to a healthy environment, life, health, food and water”. The court determined that the “fundamental rights to life and health are linked and determined by the environment and ecosystem”. The court further recognized the Colombian Amazon as subject to rights.

There are similar cases in India, Bangladesh, Colombia, Ecuador, and New Zealand. In *Modh Salim v State of Uttarakhand*, the court declared two rivers to be ‘juristic/legal persons/living entities having the status of a living person with all corresponding rights, duties and liabilities”. In 2017 the Whanganui river in New Zealand, the Ganges and Yamuan rivers in India, and the Rio Atrato in Colombia were also granted legal rights [93].

Globally, less than 15% of the land, 21% of the freshwater and 8% of the ocean are protected areas [34]. Yet, indigenous communities, which make up 5% of the global population hold 25% of the land, with 80% of planetary biodiversity. Many of the first countries to adopt rights for nature are post-colonial nations with living indigenous cultures.

In the US, which constitutes the majority of litigation cases, 70% of litigation based on federal statute is brought under the National Environmental Policy Act and Clean Air Act, which are more anthropocentric in their objectives [94]. However, it is likely that the codification of eco-laws, in the US at the local level, will bring challenging cases before the courts on the issue of personhood for nature.

#### Reconciling social and environmental justice

The trend in the growing volume of cases brought by, or on behalf of youth, introduces the concept of intergenerational equity. The UN convention on the rights of the child holds that “state parties shall undertake all appropriate legislative, administrative and other measures for the implementation of the rights recognized in the convention” [95]. In addition, the concept of intergenerational equity, is at the core of both the UN Charter and the Stockholm declaration on the environment: “the present generation has a right to use and enjoy the resources of the Earth but is under an obligation to take into account the long-term impact of its activities and to sustain the resource base and the global environment for the benefit of future generations of humankind” [96]. The right to a healthy environment is now recognized in law by more than 80 percent of United Nations Member States (156 out of 193) [97].

In the case of *Saachi v Argentina* [98], 16 children filed a petition through the UN convention on the rights of the child. The violated rights included “the right to life, right to health, prioritization of the child’s best interest, and cultural rights of petitioners from indigenous countries”. In the case of *Future generations v Ministry of the Environment* (Table 1, case 2), a group of plaintiffs aged between 7 and 26 years claimed the rights to a healthy environment, life, health, food and water. In the case of *Youth for Climate Change v EU* [99], a group of youths claimed that the impact of climate change upon them would go as far as harming their mental health.

Considering that the environmental impact will disproportionately affect certain communities, there are cases of climate litigation that have been brought by groups that include: women, climate refugees, elderly women, organic farmers, indigenous people, and civil society.

In most rulings, the courts have declined to give such groups unique standing to bring a case, for example by stating that all people will be impacted by climate change. Therefore, the strategy of framing the issue as one of disproportionate impact has not yet proven to be highly effective. However, the impact of climate litigation in generating media coverage, creating publicity, and changing public perception is evidently growing and can be expected to escalate further.

### The role of public health in climate litigation

#### Climate litigation through a public health lens

Despite growing awareness of health impacts of climate change, the public health lens is yet to be widely adopted by plaintiffs. Our study revealed that only 65 (3.96%) of the 1641 cases used a litigation strategy which included climate change at the center, with extensive use of the word “health” mentioned in the case file submitted to the court. We identified a rise in explicitly health-linked cases over recent years. This rise is consistent with the general increase in litigation cases and is not specific to litigation pursued on public health grounds.

In their study on the role of health in climate litigation, McCormick et al. (2018) found that the Clean Air Act (CAA) was used to support 32% of all cases in the US in which litigants relied on health concerns to support their claims [100]. Interviews with plaintiffs revealed that their primary incentive for pursuing cases under the CAA was that, unlike laws such as the California Environmental Quality Act and the National Environmental Policy Act, the CAA has the protection of public health as its principle goal [100]. This shows that where health is the main focus of a given legal doctrine or environmental regulation, there exists a more direct and powerful avenue for individuals to pursue litigation on the basis of health concerns.

#### Receptivity of courts to public health-framed argumentation

Amongst the 65 directly health-linked cases, there were 11 wins, 11 losses and 43 remain undetermined. The low number of directly health-linked climate cases and the number where decisions are pending, precludes accurate assessment of trends in the receptivity of courts to public health-framed argumentation. The early successes, such as Urgenda Foundation v State of the Netherlands (Table 1, case 1) and other cases are testament to the potential power of public health claims in legal argumentation, yet it remains premature to conclude whether or not public health arguments presented to courts will be seen with the credibility and weight that they warrant.

Nonetheless, one may postulate that as climate-health science matures, making clearer the links on which legal claims rest, the receptiveness of courts to public health language may rise. While McCormick et al. found that cases in which health was an issue had a win rate nearly the same as that for non-health cases (31% and 30%, respectively) [100], claims made by litigants attested to the perceived power of health risk attributions in legal argumentation. As stated, many litigants were “motivated to include health allegations in climate cases because they make the consequences of unabated climate change tangible and may increase the chances that courts will grant the relief sought by satisfying the substantive criteria needed to achieve success on the legal merits of the claim” [100]. Additionally, McCormick et al. noted that several cases in the database that centered on claims about contaminated water and used health as a central argument were won by plaintiffs seeking improved protections for local communities [100].

We contend that health ought to have a bearing on court rulings given the inherent political salience it holds. In his review of fishery management law, Craig (2014) argues that when it comes to advocating for laws targeted at ocean-focused climate change adaptation, a public health connection can provide “a political saliency and importance that is often otherwise missing” [101].

Likewise, Nosek (2018) argues that, so long as health threats in litigation cases can be proven as imminent and knowingly created, debate should naturally “shift towards the necessity of intervention by government and industry officials” [102].

#### Attribution and climate-health science

Litigation rests fundamentally upon the ability of the plaintiff to link the accused actions to evidence-backed consequences. Climate attribution science offers the ability to prove causation and is now viewed as a central ingredient for success. Contemporary use of extreme event modelling and the accessibility of this data to the court plays a large role in determining the “attributable risk” of scrutinized actions in climate litigation [103].

With climate-health science currently increasing in strength and credibility, data-backed argumentation offers powerful leverage in future health-centred climate litigation. For example, in *Neubauer et al*. *v Germany* (Table 1, case 4), plaintiffs cite IPCC data showing the risk of death, injury, and damage to health in low-lying coastal areas vulnerable to storm surges, coastal flooding, and sea level rise. Plaintiffs cited robust evidence of the link between rising allergies and lengthening pollen seasons, between respiratory diseases and both ragweed blooms and flood-induced indoor mould growth, and storm/flood disasters and psychological trauma. This is supported by the conclusion drawn by McCormick et al. (2018) that “the strength of the scientific evidence mustered to support litigants’ challenges is likely to be an important determinant of case outcomes” [100].

Cases reviewed by McCormick et al. [100] revealed that climate science was only occasionally a central part of the court decisions. This indicates the existence of competing factors which weigh into the decision-making on legal outcomes. Thus, to strengthen the relative weight placed on scientific evidence in legal decision-making on climate litigation, an argument can be made that emphasis should be put on the most scientifically sound, and socially, politically, and economically salient points, backed by expert testimony and robustly generated syntheses of the literature. An additional requirement may be for evidence to show that health risks are imminent, since it has been shown that the narrative of climate risks being distant in space and time dilutes the urgency to act now or the credibility of climate risk allegations [102].

With regards to leveraging the insights, research, and testimony of public health experts in climate litigation, it is worth noting the possibility of COVID-19 having opened a policy window for such action. Undoubtedly, COVID-19 has raised the profile of public health expertise and the value of epidemiology. Thus, public health expertise may be accorded rightful recognition in courts of law in a post-COVID-19 era, something which can and should be exploited in upcoming climate litigation.

### Economic incentives as a barrier to or incentive for public health

#### Financial liability and public health

The case law brings forward economic and financial considerations, presenting as either incentives or barriers to ‘adaptation and mitigation’, accompanied by liability for market actors. This can give rise to climate laws and policies, that are concerned with “mitigating, adapting and regulating” industrial activity which causes climate change.

Whilst the unmitigated consequences from climate change are catastrophic, a hesitance persists in some courts to “overhaul industries”, as indicated by the Juliana case [67]. Despite pledges by many corporations to reach ‘net zero’ emissions by 2050, there is not yet any contractual or legal obligation to enforce this. The agreement at COP26 to establish rules for a global voluntary carbon market also raises complex issues about the ethics of carbon offsetting, and whether access to finance comes with a human rights and health cost for local and indigenous communities in low-income countries [104].

The evolving link between financial systems, climate change, and the public health cost, is an important indicator in climate litigation [105]. It is extensively documented in the publications of UNEP(FI) [106] where “climate change is referred to by leading economists as the greatest market failure in human history”. The 2020 EU taxonomy regulation on sustainable finance, is framed along the same lines as ‘climate law and policy’, namely objectives of *Climate change mitigation*, *Climate change adaptation*, *Sustainable use and protection of water and marine resources*, *Transition to a circular economy*, *Pollution prevention and control*, *and Protection and restoration of biodiversity and ecosystems* [107] (other jurisdictions such as China have a similar, yet distinct objectives).

In the case of *Milieudefensie et al*. *v Royal Dutch Shell* (Table 1, case 9), litigants sought to extend the ruling of Urgenda to private companies. Shell is the parent company of many subsidiaries and has a principal place of business in the Hague, Netherlands. According to the summons, “the financial incentive for Shell to continue its fossil business model developed since 1890 is enormous, which is why it hampers the energy transition, and every year continues to invest dozens of billions to secure the future of the fossil industry for much of this century”. Milieudefensie campaigns for “climate-friendly banking” and believes “private banks and public asset managers are the ideal partners in that respect”. In the ruling against Shell the court stated that although “RDS cannot solve this global problem on its own. However, this does not absolve RDS of its individual partial responsibility to do its part regarding the emissions of the Shell group, which it can control and influence”.

The centrality of financial capital in global economies embeds financial institutions within the broader climate law and mitigation landscape. Financial investment is a powerful influencer on industrial and economic activities serving as an enabler, through incentives, or as a barrier. These organizations offer another avenue for communities to ensure their sentiments are reflected. With regards to financial institutions, several cases illustrate this indicator of financial liability in climate change lawsuits, in which the strategy of litigation has been referred to as “investor activism” [108].

In *McVeigh v Rest* [109], the respondent sought information from his investment fund, about any actions the respondent was taking, regarding the financial risk posed to his investment by climate change. The court decided that funds have a duty to disclose information that allow the investor to make an informed decision about the investment in relation to such climate risk. Considering the shifting values as citizens on all continents witness climate catastrophes, and the emergence of global youth movements, consumer and social preferences are changing in favor of the environment, health, and well-being. Consequentially, banks, insurers and investment funds face increasing risks of litigation if their activities are not compliant with climate frameworks.

In *JAM v International Finance Corp* [110] (IFC), a group of Indian villagers brought a case against IFC, the investment arm of the World Bank, before the US Supreme Court, because it had financed the construction of a coal fired plant in India, which polluted the surrounding area. Prior to this case, the IFC held immunity from such prosecutions, in the same manner enjoyed by foreign governments. This ruling overturned the IFC’s immunity from liability for its investments. Because the case involved the World Bank, such a ruling exemplifies how financial entities can be liable in law for financing harm to the environment and public health.

Cases of climate litigation reinforce the need for a globally coordinated approach to climate law, public health, and economics. The World Bank has offered categories for Environmental and Social Investing (ESG) including negative screening, best in class, impact investing, ESG integration, and responsible ownership [111]. All of this relies upon data provided by sovereign countries. The challenges of climate adaptation and mitigation have been translated into a new economics. This must be fully embedded in all legal systems, before massive public health devastation ensues [42]. Despite 29 of the largest 30 financial institutions having set 2050 climate goals, all 30 institutions remain committed to short terms actions, which oppose emerging sustainable finance policy, and have cumulatively enabled at least $740 billion in primary financing to the fossil fuel production value chain in 2020 and 2021 [112]. It remains likely that the financial sector will continue to enable real-economy activities misaligned with 1.5°C climate scenarios as long as they remain legally and economically viable in the short term [112].

#### Climate litigation against ‘carbon majors’ due to public health impact

Litigating against corporations so that they bear the liability for harm to public health is another means to internalize the costs that these companies are currently externalizing.

In 2020, a series of cases [113–116] were brought by US states against oil companies, using arguments similar to those used to curtail the tobacco industry for its devastating impact on public health [117]. In an ongoing case, Washington DC *(AC Racine v Exxon/BP/Shell/Chevron)* [118], the defendants are accused of “systematically and intentionally misleading the public about the central role their products play in causing climate change, one of the greatest threats facing humanity”, including through coordinating campaigns of disinformation.

In *City and County of Honolulu v Sunoco oil* [119], the claim is that not only have “Native Hawaiian cultural sites, built structures, natural resources, and infrastructure including roads, sewerage, and beach parks, and other resources been more frequently flooded and, in some cases, inundated”, but oil companies knew the climate science, and impending harm to public health since the 1950’s. The state is thus seeking financial compensation for losses, which illustrates how the courts are asked to price the impacts of climate change.

In this way market forces incentivize adaptation of corporates’ business model and capital away from harmful activity [120, 121]. The courts play a central role in deciding how quickly new legal incentives are integrated into legal systems, and these cases may become important milestones.

#### Prioritizing health above economics

Legal epidemiology is the study of law “as a factor in the cause, distribution, and prevention of disease and injury” [122]. The impact of tax and employment law on public health [123] has been studied. In the 65 public health cases included, there is an expectation from plaintiffs that government and economic actors will prioritize human health and planetary survival over economic profit. In the case of Youth Verdict v Waratah Coal, youth argue that the government has a duty of care to protect the health of its citizens. Further, in the 2020 ruling on modification to ethanol [124], the Mexican Supreme court decided an agency was restricted from raising the ethanol content in fuel, as the ‘right to a healthy environment’ must be prioritized over economic profit. Research has shown that “carbon-only” approaches to mitigating climate change that do not explicitly integrate air and water pollution and environmental justice considerations could fail to alleviate the public health harms and disproportionate burdens of fossil fuel production and use or even exacerbate them [56]. Considering that in response to the Covid-19 crisis, many (though not all) governments acted forcefully to prevent a public health emergency, including by deprioritizing the economy, it is likely that the expectation of prioritizing planetary health will only rise. With global fiscal support for the pandemic already amounting to a sheer $9 trillion by April 2020 [125], the Covid-19 crisis also further demonstrated the financial capacity for large-scale investment when imminent threats arise. At a minimum, to prevent a climate-health crisis, there is an expectation for the risk to be fully priced into the financial markets. Yet, eco-centric laws can go further. The concept of ecocide is a way of protecting the environment and health, which goes beyond civil liability, and makes it a crime to harm nature [126]. As highlighted in the 2020 status report on global climate litigation, there is mounting evidence of an increased appetite by the general public to crack down on corporate irresponsibility, requiring greater climate disclosure and an end to “corporate greenwashing” on the subject of climate and the energy transition.

## Recommendations

As the climate-health crisis further intensifies in the coming years, the expansion of legal channels for halting and prosecuting climate-destructive, health-threatening operations and anthropogenic activities will prove essential. To ensure that future legal action on environmental issues serves public health objectives, two conditions must be present: 1) public health-framed argumentation must feature strongly in allegations of wrongdoing and 2) courts are receptive to public health arguments and accord their deserved weight in the holistic assessment of cases and determination of legal outcomes. To promote such conditions, we issue the following recommendations to potential plaintiffs, public health stakeholders, climate activists, climate-engaged investors, NGOs, and other institutions:

1. Initiate or support legal cases in every jurisdiction, backed by public health science, to put pressure on governments and corporations, to upscale their commitment to de-carbonize, and uphold their duty of care responsibilities.
2. Advocate for the democratic passage of eco-centric laws and climate liability incentives, both national and international, which feature health as a primary objective; this can include the passage of an International Law of Ecocide. And interpret existing statutory requirements in a public health frame to provide a clear legal path for the pursuit of litigation according to internationally adopted legal principles of human rights and public health.
3. Mobilize additional funds for climate-health research to quantify impacts, assess attribution and assess adaptation strategies as alternative options to provide the necessary scientific evidence demonstrating the indisputable, ubiquitous, tangible, existing and imminent, and clearly anthropogenically-driven threats to human health
4. Promote greater awareness of climate-health links amongst public interest groups by incorporating dedicated climate-health curricula into higher education. Legal epidemiology, as the study of law in the cause, distribution and prevention of disease and injury, must be extended to the field of climate law to ensure legislation is instrumentalized most effectively for public health protection and promotion
5. Develop national and international environmental health law expert panels including strong public health expertise to provide expert testimony and briefings during court hearings and consult with judges on the sound interpretation and application of climate-attribution science

## Limitations

We recognize the limitations inherent in drawing conclusions based on a review of western-centric sources, which may well have failed to capture the full scope of litigation. Most importantly, legal cases from certain regions and countries are not all included in the databases searched. While nine translated climate laws and policies are reported from China, including the “National Plan for Tackling Climate Change 2014–2020”, no case litigation is reported, even though there is indication such cases have taken place in China. Similarly, three climate laws and policies are reported from Saudi Arabia including the “Saudi Arabia Vision 2030” and 26 from Vietnam, yet no cases are reported for either country.

Aside from the countries and regions for which case laws have not been included, there may also have been criminal and penal charges (i.e. criminal litigation) not captured given the Western-centric focus on civil litigation within these databases. The sources used are also both English language databases, respectively from the United States and the United Kingdom. The Common Law system underpinning the reported cases also has roots in the British Empire [127], introducing an inevitable bias in the legal scope of cases captured in this review.

Finally, we recognize that our “case definition” of public health-linked cases may be too narrow to capture all those cases which truly express a concern for human health as one of the many issues at stake in climate litigation. Indeed, we observed a far greater number of cases which alluded to aspects of public health under threat without explicitly using the word “health”. The relative importance of these cases should not be overlooked, for they undoubtedly represent a shifting mindset towards climate change, with the perceived threats to human health taking a more central role.

Climate Litigation is restricted by barriers preventing access to justice, including access to legal knowledge. We did not use subscription case law sources, including Westlaw, Thompson Reuters, and Lexis Nexus, RELX, as these databases are not open access and a costly subscription can limit access for prospective climate litigants especially in low-income countries. Perhaps, restricted access to legal sources exacerbates the problem of climate injustice, as it is a reality that those disproportionately impacted by climate change have least access to justice.

We have not examined questions of jurisprudence, which extend into theoretical questions such as what is law? Yet, climate Science can undermine the legitimacy of legal systems, as if laws are not aligned with science *should* they be valid law. We researched the field of climate law through the lens of legal cultures, which have caused climate change, and so the western, anthropocentric, legal culture is reflected in its research.

## Conclusion

Despite its limitations, this relatively comprehensive review of climate litigation over the past three decades has revealed notable developments in the legal field of environmental governance, many of which are encouraging from public health, human rights, and eco-centric perspectives. The field is undergoing a rapid transition.

Yet reticence persists. Defence teams can no longer argue against the validity of the science, or ignorance of the link between anthropogenic climate disruption and human health harm. In the context of climate health emergency, it is evident that amplification of decarbonisation efforts is urgently required. Litigation offers a powerful tool to drive multi- sector transition. Given the shortcomings that remain in the widespread use and acceptance of public health claims in legal argumentation used for litigation, we advise that laws and legal incentives are created, which position public health as a central focus. In the interests of future generations, we argue for more funding to be mobilized towards climate-health evidence and further develop this research as a scientific discipline, the promotion of greater public and industry awareness of climate-health links to support more widespread utilization of public health claims in allegations of wrongdoing, and that a legal panel of climate-health experts be devised which can be called upon by plaintiffs to provide expert testimony and deliver credible yet compelling research briefings during court hearings.

We advocate for the establishment of multi-lateral body, with legally binding power, to access breaches of existing law, identify laws that amplify disproportionate health impacts upon specific communities, responds to systemic racism, corruption, and inequities within legal systems, is aligned with the Hague to bring criminal charges against nations and corporations, and works to develop real legal incentives to ensure planetary and human safety.

Considering the impacts of climate change, we must ensure that health risks are fully integrated into the economic cost should companies seek to avoid liability for climate risks. Should this recommendation be adopted, we are confident we will see further developments in the field of litigation where public health arguments play a key role in mobilizing legislative action. As the risk of harm to public health is integrated into the economy, through legal liability, this can incentivize a well-being/health-based economy. This will be a win for not only the plaintiffs pursuing litigation, but also for the whole of humanity whose health and future survival is under dire threat by the climate crisis.

## Supporting information

S1 Data (XLSX)

## Abbreviations

IPCC: International Panel on Climate Change
CLL: Climate Change Litigation
